# Basic self-knowledge and transparency

**DOI:** 10.1007/s11229-016-1235-5

**Published:** 2016-10-06

**Authors:** Cristina Borgoni

**Affiliations:** Department of Philosophy, University of Graz, Heinrichstrasse 26/V, Graz, 8010 Austria

**Keywords:** Transparency model, Privileged self-knowledge, Basic self-knowledge, Cogito judgment

## Abstract

*Cogito*-like judgments, a term coined by Burge (1988), comprise thoughts such as, *I am now thinking*, *I [hereby] judge that Los Angeles is at the same latitude as North Africa, *or *I [hereby] intend to go to the opera tonight.* It is widely accepted that we form *cogito*-like judgments in an authoritative and not merely empirical manner. We have privileged self-knowledge of the mental state that is self-ascribed in a *cogito*-like judgment. Thus, models of self-knowledge that aim to explain privileged self-knowledge should have the resources to explain the special self-knowledge involved in *cogito* judgments. My objective in this paper is to examine whether a transparency model of self-knowledge (i.e., models based on Evans ’ 1982 remarks) can provide such an explanation: granted that *cogito* judgments are paradigmatic cases of privileged self-knowledge, does the transparency procedure explain why this is so? The paper advances a negative answer, arguing that the transparency procedure cannot generate the type of thought constitutive of *cogito* judgments.

## Introduction

According to Burge (1988, 2011), *cogito*-like judgments (hereafter ‘*cogito* judgments’ for short), are paradigmatic examples of self-knowledge acquired in a direct, authoritative, and not merely empirical manner. Examples of this type of mental self-ascription include, I am now thinking, I [hereby] judge that Los Angeles is at the same latitude as North Africa, or, I [hereby] intend to go to the opera tonight.^1^ Despite disagreements about their specific epistemic traits, it is widely accepted that, if we have privileged self-knowledge at all—i.e., knowledge of one’s own mental states that is epistemically different from and superior to the knowledge of others’ mental states—, *cogito* judgments are prime examples.^2^

One model of self-knowledge that is extensively discussed in recent years is the transparency model. Transparency models are based on Evans’ (1982) remarks suggesting that an explanation of self-knowledge is to be found in relation to the so-called transparency procedure—i.e., answering the question whether we believe that *p* by answering the question whether *p*. Transparency models of self-knowledge hold that instances of privileged self-knowledge are formed (or achieved) by the application of the transparency procedure.

The guiding question of this paper is the following: granted that *cogito* judgments are paradigmatic cases of privileged self-knowledge, does the transparency procedure provide an explanation of why this is so?^3^ The compatibilist debate between anti-individualism and self-knowledge during the late eighties and nineties discussed *cogito* judgments extensively. However, the subsequent debate that centered exclusively on self-knowledge has shifted its attention to other types of second-order judgments, such as self-ascriptions of standing beliefs. Whether transparency models explain how *cogito* judgments are instances of privileged self-knowledge remains an open question.

The paper develops as follows. Section 2 explains Burge’s notion of *cogito* judgments and identifies the main psychological characteristics of this type of thought. Section 3 explains the core claims of transparency accounts of self-knowledge and discusses the first set of criticisms of such accounts. Section 4 advances the main criticism of transparency models: namely, that the application of the transparency procedure does not produce *cogito* judgments. Since transparency models aim to explain privileged self-knowledge by explaining how the correspondent mental self-ascription is formed via the transparency procedure, I conclude that such models fail to explain how *cogito* judgments stand as privileged self-knowledge. Section 5 summarizes the paper’s arguments and briefly discusses their scope of application.

## *Cogito* judgments

Burge has coined the term ‘*cogito* judgments’ after Descartes’ *cogito* (I am now thinking) to refer to a particular class of second-order judgments that represent what he then called ‘basic self-knowledge’. When he first discussed such a class of thoughts in his (1988), Burge’s objective was to argue that anti-individualism is compatible with privileged self-knowledge.^4^ Burge has advanced an explanation of why we have privileged self-knowledge of *cogito* judgments in later works (see for example his 2011). For the purposes of this paper, it is not necessary to discuss whether Burge’s view on compatibilism has succeeded, nor is it required to agree with Burge’s explanation of self-knowledge.^5^ What matters for this paper is the nature of those thoughts that Burge individuated when he termed the notion *cogito* judgments. I explore the nature of such thoughts in the remainder of this section.

Let us start with a few examples. Consider the following thoughts:

1. I am thinking.
2. I [hereby] judge that there will be no third world war.

Thought (1) is Descartes’ *cogito*. Thoughts like (2) have many similarities to thought (1), and thus can legitimately be called ‘*cogito*-like judgments’ (see Burge 1988). Both thoughts (1) and (2) present the following specific characteristics: (i) they are a type of *judgment*: they are occurrent thoughts with associated truth conditions; and (ii) they are a type of *mental self-ascription*: when making the judgment, the individual self-ascribes a mental predicate of a particular type, namely, a *mental occurrence* of the individual who makes the judgment. Trait (ii) is particularly important to distinguish proper cases of *cogito* judgments from other types of mental self-ascription that are not *cogito* judgments, such as the following thought:

(3): I believe that there will be no third world war.

Thought (3) is a judgment by which the individual self-ascribes a mental predicate. However, assuming that thought (3) self-ascribes a standing belief, it does not display features (ii). The subject matter of (3) is a mental *state* of the individual who makes the judgment. However, according to feature (ii), a *cogito *judgment is about a mental *occurrence* taking place while the individual performs the judgment. Being about one’s own mental states is not sufficient to satisfy trait (ii). *Cogito* judgments concern present occurrences (Burge 2011, p. 209).^6^

According to Sawyer (2002), failure to see why thought (3) is not a *cogito* judgment is the basis of many misplaced criticisms of Burge’s compatibilist thesis.^7^ She presses on this issue in her defense of Burge’s views:

what distinguishes the *cogito*-like self-attributions from the non-*cogito*-like self-attributions is that in the latter case there are two distinct mental states—one standing, and the other occurrent. The *cogito*-like self-attributions, in contrast, are such that the first-order thought and the second-order judgment occur in the same mental act. (Sawyer 2002, p. 123)

As Sawyer highlights, the fact that a given mental self-attribution has two independent components shows that it is a not *cogito* judgment. Thought (3), for instance, is composed by both the mental act of judging that one believes *p*, and the belief state itself. However, a mental self-ascription composed by two occurrent mental states—as opposed to one occurrent and another standing—does not guarantee it to be a *cogito* judgment. Because both mental occurrences might be independently thought, they might not be part of the *same mental act*. Traits (i) and (ii) are insufficient to identify a *cogito* judgment. Consider the following case:

Imagine that someone, let’s say Paul, suddenly pays attention to his own reflection in the mirror. Based on the perception of his expressions of consternation, and knowing what such expressions mean in his case (but without introspecting on his feelings), Paul judges, I am thinking, again, of my pitiful life! If this is a possible case, it is a case in which a mental occurrence is correctly self-attributed (Paul is in fact thinking of his pitiful life), but such a mental occurrence is not thought within the same mental act that his mental self-ascription is thought. Paul’s self-ascriptive judgment is about one of his mental occurrences. However, the judgment and its subject matter are independent mental occurrences: they are merely simultaneously occurrent in Paul’s mind. His mental self-ascription is not a *cogito* judgment.

Therefore, if someone forms the following thought in a situation analogous to Paul’s, this person’s thought is not a *cogito* judgment:

(4): I judge that there will be no third world war.

Thus, even if an individual were to self-ascribe an occurrent mental state, the thought would not be a *cogito* judgment if the individual were not *thereby* (as a result of having the thought) *in* the ascribed occurrent state. As Paul’s example shows, there are thoughts that might exhibit trait (ii)—be self-attributions of mental occurrences—but still be composed of independent mental occurrences. Thus, a further trait is required to identify *cogito* judgments. *Cogito* judgments:

iii. *have a dual-level component: *the second-order judgment and the ascribed mental occurrence both are part of a single thought. The self-ascribed mental occurrence is thought *in* and *through* the performance of the second-order judgment.

Trait (iii) is an essential feature of *cogito* judgments, which distinguishes them from other types of judgments containing self-ascription of mental occurrences. The term ‘in’ in the characterization of trait (iii) captures the relation of the first- and second-order thoughts to each other: the first-order thought is contained *in* the second-order thought. The term ‘through’ describes how first- and second-order thoughts are related by an individual’s act of thinking—that is, an individual thinks the first-order thought *through* the act of thinking the second-order one.^8^ Both mental occurrences—of thinking and of judging that one is thinking— are part of the same mental act. In a *cogito* judgment, the individual both *thinks* the first-order mental occurrence and *judges* that she is thinking the thought in the *same thought*.

With trait (iii), we can distinguish between thoughts (2) and (4) more clearly. The former thought exhibits trait (iii); the latter, however, does not. Such a difference between the two thoughts is represented by the presence or absence of ‘[hereby]’ in the thought’s content. However, the inclusion of ‘[hereby]’ in the thought’s content does not mean that the individual herself employs the notion of ‘hereby’ when forming a *cogito* thought. The term ‘hereby’ represents the fact that in a *cogito* judgment, the subject matter and the judgment are part of the same thought.

Features (i)–(iii) identify *cogito* judgments as a distinctive type of thought by describing the *psychological* traits of such thoughts. *Cogito* judgments also present some distinctive *epistemological* traits, some of which are related to their psychological features. As mentioned previously, the epistemic thesis that *cogito* judgments count as privileged self-knowledge is widely accepted. According to a less prevalent but still relatively popular thesis, *cogito* judgments are self-verifying.^9^ The latter thesis is central to Burge’s notion of *cogito* judgments. I will expand on it in the remainder of this section.

Burge holds that *cogito* judgments are special cases of privileged self-knowledge since they are self-verifying. However, Burge distinguishes between *pure cogito* judgments and *impure cogito* judgments based on their self-verifying aspect. According to Burge, only pure *cogito* judgments are *logically self-verifying*. Impure *cogito *judgments are performatively self-verifying.^10^ Pure *cogito* judgments are self-verifying due to their self-referential form (Burge 1988, 2011; Sawyer 2002, p. 109). Take as examples of pure *cogito* judgments, thought (1) and the thought, I am thinking that writing takes time. It holds for both thoughts that:

by [their] reflexive, self-referential character, the content of the second-order judgment is logically locked (self-referentially) onto the first-order content which it both contains and takes as its subject matter. (Burge 1988, pp. 659–660).

The logic and meaning of the intentional thought content requires that if the content is thought, it is true (Burge 2011, p. 214).

Pure *cogito *judgments are thus infallible. ‘If one thinks a thought of that form, one thinks a truth’ (Burge 2011, p. 208).

Impure *cogito* judgments are not logically self-verifying due to their form. Instead, impure *cogito* judgments are *performatively self-verifying* (Burge 2003, p. 124). These are *cogito* judgments where ‘the intentional content does not strictly entail that a thinking of the thought [is] self-verifying, or even true’. We could conceive a case where an individual thinks, I [hereby] judge that there will be no third world war, but fails to *judge* that there will be no third world war. According to Burge, such cases are abnormal, but are possible. Although normal occurrences of *cogito* judgments are performatively self-verifying, they are not immune to error. In his words:

In cases of reflexive performatives that are not pure *cogito *cases—like I hereby judge that Socrates drank some hemlock or I hereby intend to give to Oxfam—the self-verification is not formally guaranteed merely by thinking the thought. But their truth is guaranteed, in normal circumstances, by the clearly understood performance of the act. (Burge 2003, pp. 135–136)

In impure *cogito *cases (e.g. I [hereby] judge that writing requires concentration), the indicated lower-level mode (e.g. judgment) is not entailed to be the mode of the content cited by the underlining. In such a *cogito *thought, one could judge that one is judging the lower-level content, and be mistaken about one’s making a judgment. [...]. Such cases are pathological misuses of the *cogito *form of thought. (Burge 2011, p. 208)

The thesis that *cogito* judgments are self-verifying, either logically or performatively, has been challenged during the compatibilist debate (see Gallois 1996; Bernecker 1996 and Goldberg 2000). Even so, nowadays many philosophers of self-knowledge seem to accept such a thesis.^11^ In any case, what matters for this paper’s arguments is the idea that *cogito* judgments are a particular class of mental self-ascriptions that present certain specific psychological features and are epistemically special. So, for my purposes, Burge’s distinction between pure and impure *cogito* judgments can be understood in a schematic way as follows:

In pure *cogito* judgments, there is no specification of the type of mental occurrence that is the judgment’s subject matter. Neither thought (1) nor the thought, I am thinking that writing takes time, specifies the type of mental occurrence its thinker is in. In contrast, an impure *cogito* judgment specifies a mode of thinking as part of that judgment’s content. In thinking thought (2), the individual self-ascribes a judgment. But an impure *cogito* judgment could be about any other type of mental occurrence. Although *cogito* judgments concern present mental occurrences, such occurrences themselves need not be judgments (Burge 1996, 2003). The following is another example of an impure *cogito* judgment:

(5) I [hereby] intend to go to the opera tonight.

This paper takes pure *cogito* judgment (1) and impure *cogito* judgments (2) and (5) as its primary working examples. Any unified model of self-knowledge that aims to explain privileged self-knowledge should be able to explain how an individual has the privileged self-knowledge expressed by these three thoughts.^12^ I turn next to the question of whether a transparency model of self-knowledge can explain how *cogito* judgments (1), (2), and (5) stand as privileged self-knowledge by examining whether such thoughts can be generated by the transparency procedure.

## Transparency models of self-knowledge

Transparency models are based on Evans’ (1982) remarks on self-knowledge, as exemplified by the following much-cited passage:

I get myself in a position to answer the question whether I believe that *p* by putting into operation whatever procedure I have for answering the question whether *p*. [...] If a judging subject applies this *procedure *[my emphasis], then necessarily he will gain knowledge of one of his own mental states (Evans 1982, p. 225).

According to the mentioned procedure for gaining self-knowledge, one answers the question of whether one believes that *p* by answering the question, whether *p*. This procedure has been called the ‘transparency procedure’ (Byrne 2005, 2011a). Alternatively, the condition met by the procedure has been called ‘the transparency condition’ (Moran 2001).^13^ A transparency model is built on the idea that there is an epistemically relevant relationship between the two questions and corresponding answers that compose the transparency procedure. The application of the transparency procedure, it is argued, yields self-knowledge.

Evans (1982) has explicitly given a very brief treatment of self-knowledge; he has not pursued a *model* based on transparency. However, many inspired by Evans—e.g., Moran (2001), Byrne (2005, (2011a), Boyle (2011), Fernández (2003, (2013), and others—have formulated proper models of self-knowledge. In what follows, I first explain Byrne’s transparency model and raise the first set of criticisms against it. After that, I discuss my main and general criticism of transparency models: namely, that the transparency procedure does not generate *cogito* judgments.

### Byrne’s model and the first set of criticisms

Cassam (2015) suggests that the most natural and reasonable interpretation of the transparency procedure is one according to which its application generates indirect mental self-ascriptions. The transparency procedure regards a relation between answers to two different questions: a question about *p* and a question about whether one believes that *p*. Thus, it is natural to think that the transparency procedure encompasses a transition between an answer to the former question and an answer to the latter. If this is so, then applying the procedure generates indirect self-knowledge—and it is reasonable to suppose that the aforementioned transition is inferential. In Cassam’s words:

How can the self-knowledge acquired by putting into operation a procedure for answering a different question be anything other than indirect? There must be some kind of transition from answering the question whether *p* to answering the question whether you believe that *p*, but knowledge that is genuinely direct would involve no such mental transition. (Cassam 2015, p. 4)

Cassam (2014, (2015), who does not defend transparency models, holds that a lot of self-knowledge is indirect and inferential. What is puzzling, he seems to suggest, is the insistence that, nevertheless, a transparency model can generate direct, non-inferential self-knowledge.

However, though Byrne (2005, (2011a) supports a transparency model, he is not committed to such a puzzling position.^14^ According to Byrne (2011a), the transparency procedure consists in inferential reasoning from a premise about the world to facts about one’s own mind. In reference to Evans’ famous example, Byrne suggests that an individual is able to self-ascribe the belief that there is not going to be a third world war via an inference from his own judgment that there is not going to be a third world war. Byrne specifies this procedure in terms of the ‘doxastic schema’ (2011a), a rule that we apply to gain self-knowledge:^15^

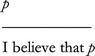



According to Byrne, following the doxastic schema yields self-knowledge because the inference involved in the schema has certain qualities that warrant its conclusion. He defends the doxastic schema as strongly self-verifying: ‘if one reasons in accordance with the doxastic schema, and infers that one believes that *p* from the premise that *p*, then one’s second-order belief is true, because inference from a premise entails belief in that premise’ (Byrne 2011a, p. 206).

The special characteristics of the doxastic schema, according to Byrne, explain *privileged access* and *peculiar access,* two key elements to be explained by models of self-knowledge, cf. Byrne (2011a, p. 202). Byrne characterizes *privileged access* as the higher probability that first-personal beliefs have to amount to knowledge in comparison to beliefs about others’ mental states. If the doxastic schema is strongly self-verifying, its application has a high probability of generating knowledge. *Peculiar access*, according to Byrne, refers to the difference between the methods used to gain self-knowledge and those used to gain knowledge of other people’s minds. The doxastic schema only works in the first-person case: ‘Inferring that André believes that *p* from the premise that *p* will often lead one astray’ (Byrne 2011a, p. 207).

Several criticisms have been raised in response to Byrne’s model. Many of these criticisms target the quality of the inference employed in the doxastic schema. For example, Boyle (2011) points out that this inference is neither deductively valid nor inductively strong. Valaris (2011) objects that, in contrast to any other rule of inference, the doxastic schema’s inference cannot be deployed in hypothetical reasoning. In hypothetical reasoning, the individual need not believe in the premise in order to draw an inference. The individual only needs to suppose the premise to be true. However, the doxastic schema only works if the individual believes in the premise.^16^

My first criticism of transparency models does not target the quality of the inference involved in the transparency procedure as conceived by Byrne. Instead, my criticism targets the very fact that the transparency procedure generates indirect or inferential self-knowledge. According to Burge, *cogito* judgments amount to non-inferential self-knowledge. They are non-inferentially formed and their epistemic warrant does not rely on inferences. Thus, given Byrne’s model (and possible variants of it), the transparency procedure cannot explain how an individual forms *cogito* judgments. In what follows, I expand on this argument. I refer to models that are based on transparency and that hold that a belief self-ascription involves an inference from the individual’s take on *p* as ‘Byrne-like transparency models’.

Trait (iii) is a distinctive aspect of *cogito* judgments (see Sect. 2). *Cogito* judgments are self-reflexive and have a dual-level component, in that the individual thinks the first-order mental occurrence in and through judging that she does so. According to Burge, this psychological aspect of *cogito* judgments suffices to make the self-ascription a non-inferential one, at both etiological and epistemic levels. Burge says:

The structure of these judgments [pure and impure *cogito* judgments] shows clearly that they are not inferential. When I judge I am thinking, or I [hereby] judge that Brahms is greater than Chabier, or I intend to take the metro, the judgment is reflective in a way that precludes individual-level inference. The judgment about the psychological state has, as a constituent part, the psychological state instance that the judgment is about. Judgment and subject matter are part of a single thought. (Burge 2011, p. 184).

According to Burge, the dual-level component aspect of *cogito* judgments evinces that the thought has not been inferentially formed. Since the first-order level thought—e.g., thinking or judging that *p*—is thought through its self-ascription, the first-order thought could not be used by the person as evidence, in an inferential reasoning, to conclude that she is thinking so. This is the reason why Burge says that the reflective aspect of a *cogito* judgment precludes individual-level inference.^17^ In cases of *cogito* judgments, the individual does not first grasp that she is thinking, and then conclude that she is in such a mental state. Nor does the person think the first-order thought, and then judge that she is thinking such a thought. Both thoughts are part of the same mental act.

According to Burge, a *cogito* judgment is not *inferentially* warranted either. As briefly discussed in Section (2), Burge holds that *cogito* judgments are self-verifying. If someone thinks a pure *cogito* judgment, she thinks a truth. In cases of impure *cogito* judgments, there is some room for mistakes, if only very little (namely, only in cases of psychological abnormalities). Being self-verifying is arguably one epistemic aspect of *cogito* judgments due to their psychological form. Thus the nature of *cogito* judgments shows that they stand as non-inferential self-knowledge (whatever one’s epistemic explanation of this type of warrant might be).^18^

Byrne-like transparency models generate inferential mental self-ascriptions that allegedly stand as self-knowledge. The mental self-ascriptions generated by the application of the transparency procedure are inferences from a premise about the world to a conclusion about one’s mind. However, the traits that identify the type of thought correspondent to a *cogito* judgment also imply that a *cogito* judgment is non-inferentially formed or warranted. Thus, Byrne-like transparency models cannot explain privileged instances of *cogito* judgments. And if Cassam’s suggestion is accurate—if the only reasonable way to make sense of the transparency procedure is by understanding it as generating indirect, inferential self-ascriptions—then the only conclusion is that transparency models in general cannot explain how *cogito* judgments stand as instances of privileged self-knowledge.

One way to reply to the above argument is by resisting Cassam’s suggestion (even if one accepts that Byrne’s model cannot explain how *cogito* judgments count as privileged self-knowledge). As a matter of fact, there are models of transparency intended to be non-inferential. Thus, the previous argument does not seem to hold as a general criticism of the explanatory power of transparency models regarding *cogito* judgments.^19^ *Pace* Cassam, one could insist that the application of the transparency procedure does not generate inferential self-knowledge. Works by Moran (2001), Boyle (2011), and Fernández (2003, 2013) advance this conception of the transparency procedure.

Moran (2001), for example, advances a non-inferentialist picture of self-knowledge that incorporates transparency. He suggests that, ‘a statement of one’s belief about X is said to obey the Transparency Condition when the statement is made by consideration of the facts about X itself’ (Moran 2001, p. 101). Moran holds that such a condition relates to self-knowledge because in considering the reasons for X, one makes up one’s mind about X. Deliberation is a central element in Moran’s view of self-knowledge. Moran does not explicitly specify the nature of the transition between the two answers that compose the transparency procedure. But his proposed idea suggests that in deliberating whether *p*, and thereby making up one’s mind about *p*, one gains knowledge of one’s own thoughts regarding *p*.^20^

Given the existence of models that, like Moran’s, aim to be non-inferential, the argument presented above cannot conclusively settle the question driving this paper: namely, whether a transparency account of self-knowledge can explain how *cogito* judgments amount to privileged self-knowledge. In what follows, I turn to a general criticism of the transparency model that targets both inferentialist and non-inferentialist interpretations of the transparency procedure. My criticism targets the idea shared by any transparency model: the idea that we self-ascribe a belief whether *p* by answering the question whether *p*. I argue that the application of the transparency procedure does not generate thoughts with the dual-level component trait (iii), and thus, does not generate *cogito* judgments.

## Second set of criticisms

The core idea shared by any transparency model of self-knowledge is that privileged self-knowledge is achieved, or formed, by the application of the transparency procedure. The procedure says that we answer the question whether we believe that *p* (i.e., we self-ascribe beliefs) by answering the question whether *p*. Applying such a procedure, it is claimed, yields privileged self-knowledge. Let me first raise a series of worries for transparency accounts regarding their capability to generate *cogito *judgments via such a procedure.

One immediate difficulty for transparency models to generate a *cogito* judgment is that the transparency procedure traditionally concerns self-ascription of standing beliefs.^21^ However, as discussed in Sect. 2, *cogito* judgments concern mental occurrences. Thoughts of the form (3) I believe that there will be no third world war are not *cogito* judgments. A *cogito* judgment needs to be about an occurrent mental thought: e.g., (2) I [hereby] judge that there will be no third world war. Thus, in its original form, the transparency procedure cannot generate *cogito* judgments—including the working examples (1), (2) or (5)—since each of them self-ascribes mental occurrences.

One could reply, however, that the transparency procedure can be easily modified to account for the self-attribution of occurrent mental states, like judgments, as well as of standing beliefs.^22^ For example, one modified version of the transparency procedure might take the following form: we answer the question whether we *judge* whether *p* by answering the question whether *p*. In fact, some criticisms of the transparency procedure seem to support this kind of alternative. Such criticisms suggest that in answering the question whether *p*, one judges whether *p*; thus, when applying the transparency procedure, the individual does not gain knowledge of a standing belief (a belief that perhaps the individual wanted to know about in the first place). Rather, she gains knowledge of a newly formed state—a judgment, for example (see Gertler 2011b; Lawlor 2003). One could, thus, insist that if the transparency procedure explains anything at all, it only explains the kind of self-knowledge involved in self-ascription of judgments. And this is sufficient to generate self-knowledge involved in *cogito* judgments.

However, such a modification of the transparency procedure is insufficient to explain the formation of *cogito* judgments. Self-ascription of a judgment is sufficient only to generate thoughts like (4) I judge that there will be no third world war. However, if (4) does not have a dual-level component, then thought (4) is not a *cogito* judgment. (I explored the difference between thoughts (2) and (4) in Sect. 2.)^23^ Moreover, a *cogito* judgment does not merely involve self-ascription of an occurrent *judgment*. Self-ascription of a judgment is necessary to generate thoughts like (2) I [hereby] judge that there will be no third world war, but it is not necessary to generate thoughts like (1) and (5).

*Cogito* judgments can be about many different types of mental occurrences. Thus, as discussed, the modification of the transparency procedure in terms of self-ascription of judgments clearly does not generate *cogito* judgments about other types of mental occurrences. Still, one could insist, further modifications of the transparency procedure are possible in order to generate more diverse kinds of thoughts. Before I turn to my general criticism of transparency, I briefly discuss some further worries related to such a maneuver. For that, let us recall the three working examples of *cogito* judgments:

- (1) I am thinking.
- (2) I [hereby] judge that there will be no third world war.
- (5) I [hereby] intend to go to the opera tonight.

Consider thought (5). In order to generate such a thought, the transparency procedure would need to be such that it could prescribe the self-ascription of an intention merely via consideration of the intention’s subject matter. However, it is unclear how one could successfully do this. It is unclear what kinds of considerations about the intentions’ subject matter—other than intending to $\upphi$ itself—could possibly generate a corresponding intention that is then self-ascribed by the individual.

One might insist that considerations on the desirability of doing *p* constitute a good candidate for reformulating the transparency procedure regarding intentions. However, deciding whether a given course of action is desirable does not clearly generate an intention towards *p*. Deliberating whether it is desirable to take a break now does not need to generate an intention to take a break now. Thus, settling this issue would not shed any light on whether I intend to take a break now, since it is possible that no intention has been formed in the first place. Although not impossible, modifications to the transparency procedure do not seem to apply very well to self-knowledge involved in *cogito* judgments about occurrent intentions.

Next, consider thought (1). A possible modification to the transparency procedure in order for it to generate the mental self-ascription involved in (1) might have the following form: we answer the question whether we are thinking by answering the question whether *p*.^24^ Such a procedure might generate self-knowledge; an individual who is answering the question whether *p* is thinking. However, this is a rare and unlikely version of the procedure: after all, our knowledge of our mental activity is more fine-grained than merely knowing that we are thinking when we are carrying on different mental activities. Furthermore, it is not obvious that the generated self-ascriptions are in fact *cogito* judgments. In what follows, I raise my general criticism and argue that such self-ascriptions are not examples of *cogito* judgments. The application of the transparency procedure, independent of its various versions, does not clearly generate thoughts with a dual-level component feature.

A *cogito* judgment is a thought that the individual thinks in the following way: she thinks the ascribed thought (e.g., an occurrent judgment, an occurrent intention, or the mere activity of thinking) in and through judging that she does so. This is a specific aspect of *cogito* judgments captured by trait (iii). The transparency procedure, however, does not predict that the thought produced by the application of the procedure has such a characteristic, nor does the procedure seem to have resources to do it.

In Sect. 3.1, based on Cassam’s observations, it was suggested that the most plausible understanding of the transparency procedure is the one that conceives it as generating indirect, inferential mental self-ascriptions. However, if this is so, such a mental self-ascription is not a *cogito* judgment (see also Sect. 3.1). The form of a *cogito* judgment precludes it from being inferentially formed. The self-reflexive character of a *cogito* judgment, captured by trait (iii), precludes individual-level inference. The individual thinks the first-order thought by performing the judgment about that thought.^25^ In contrast, the inferential reading of the transparency procedure seems to suggest that an individual first thinks the first-order thought and only later ascribes that thought to herself. However, in a *cogito* judgment, the individual thinks both thoughts in the same mental act. The inferentialist version of the transparency procedure fails to produce *cogito* judgments.

On the non-inferentialist version of the transparency procedure, the generated mental self-ascription does not seem to present trait (iii) either. The application of the transparency procedure seems to indicate that in self-ascribing a given mental predicate, the individual thinks *only* about the subject matter of her first-order thought. If there is no transition between the two answers involved in the transparency procedure, the individual thinks about *p* when self-ascribing mental state *S*.^26^ But thinking only about the subject matter of one’s ascribed thoughts (i.e., only thinking about *p*) is insufficient for a thought to be a *cogito* judgment. A *cogito* judgment is a thought that involves thinking also about the self-ascribed mental occurrence.^27^

This interpretation of the type of thought generated by the application of the transparency procedure, if understood non-inferentially, seems to be in line with Evans’ remarks. When Evans briefly discusses self-knowledge, his main motivation is to conceive an alternate picture of self-knowledge to the so-called Cartesian picture. He explicitly aims to avoid the inner observation picture of self-knowledge. He then suggests that, ‘in making a self-ascription of belief, one’s eyes are, so to speak, or occasionally literally, directed outward—upon the world’ (Evans 1982, p. 225). This much-cited passage has fed many intuitions underlying transparency models.

However, ‘directing one’s eyes outward’ does not seem to be enough to give us the type of thoughts that consist in *cogito* judgments. *Cogito* judgments require that the individual’s eyes are, so to speak, directed both outwards and inwards simultaneously. In a *cogito* judgment, one not only thinks the thought that is ascribed; one also thinks the very self-ascription. In this sense, in the attempt to avoid the inner observation view of self-knowledge, Evans has apparently left us without resources to understand *cogito* judgments. Denying that knowing one’s own thoughts is a type of inner perception does not require the denial that one can direct one’s eyes inwards. This is so precisely because one can conceive self-knowledge outside the (inner-outer) sensory approach to self-knowledge. Directing one’s eyes inwards or outwards is just a metaphor. But if one wants to insist on the metaphor, one needs to appeal to both inward and outward directions in order to accommodate the dual-level component presented by *cogito* judgments. Non-perceptual models of self-knowledge can be compatible with the idea that we can form *cogito* judgments and that they stand as privileged self-knowledge.^28^

## Concluding remarks

This paper has argued that transparency models of self-knowledge cannot explain how *cogito* judgments stand as instances of privileged self-knowledge. Transparency models predict that mental self-ascriptions that amount to privileged self-knowledge are produced by the application of the transparency procedure. However, the transparency procedure cannot generate *cogito* judgments. On an inferentialist understanding of transparency, mental self-ascriptions are inferentially generated. However, the form of *cogito* judgments precludes them from being inferential. Moreover, the inferential model seems to predict that the individual first thinks the first-order level thought and only later thinks the self-ascription, which does not match the dual-level component feature of *cogito* judgments. On the other hand, in a non-inferentialist picture of transparency, the transparency procedure does not seem to generate thoughts with a dual-level component either. In self-ascribing a mental state via the application of the transparency procedure, the individual thinks only the ascribed state. However, it is part of what identifies *cogito* judgments that they have a dual-level component. In a *cogito* judgment, the individual thinks the first-order thought in and through thinking that she thinks the ascribed thought.

The conclusion of this paper’s argument raises a criticism of transparency models of self-knowledge that targets their explanatory power. It concludes that such models cannot explain the formation of *cogito* judgments, and thus cannot explain one particular class of privileged self-knowledge. One might raise doubts, however, about the importance of such results. One might insist that this conclusion is too narrow to deserve attention: *cogito* judgments just add to the long list of mental states that do not succumb to transparency, which is not particularly surprising. One might even insist that transparency theorists would emphasize that they did not intend their theory to cover all self-knowledge. Thus, it should not be a problem that *cogito* judgments are excluded from their models.

Such attempts to resist the results achieved by this work, however, are misguided. On the one hand, showing the explanatory limits of a given model is a relevant result, no matter if the model has other limitations that have already been pointed out. If transparency models are unable to explain the self-knowledge involved in *cogito* judgments, this result *per se* deserves attention. Such models are still very influential. On the other hand, if one insists on the idea that transparency models have never intended to give a unified model of self-knowledge that includes *cogito* judgments (see ft. 12), then we should be provided with some evidence that this is in fact the case. However, there is no explicit delimitation of transparency models in relation to *cogito* judgments. Furthermore, there are some transparency models, which do aim at a broad explanation of self-knowledge such as the models developed by Byrne (2005, (2011a, (2011b) and Fernández (2003, (2013).

Additionally, there is an independent (and perhaps stronger) reason to include *cogito* judgments within the items to be explained within our theories of self-knowledge. As I mentioned earlier, *cogito* judgments are accepted to be paradigmatic cases of privileged self-knowledge.^29^ Moreover, for many philosophers, understanding the type of self-knowledge involved in *cogito* judgments is crucial for our understanding of the general phenomenon of self-knowledge. Descartes, whose reflections were based on the *cogito*, is among such philosophers. Burge is another example. According to Burge (2013), understanding the traits of *cogito* judgments and the cognitive capacities required for and employed in the formation of such judgments is part of the general explanation of our capacity for self-knowledge. This picture is reflected in Burge’s earlier writings when he calls this class of judgments ‘basic self-knowledge’. And more recently, it is elaborated as follows:

Although *cogito* cases are very special cases, they contain, in germ, many of the key features that are central to and constitutive of all types of authoritative self-knowledge. *Cogito* instances are special and peculiar in their self-evidence, self-justification, and self-verification. But they point beyond themselves in other respects: their non-inferential immediacy; their first-person character; their use of canonical specification of contents that requires understanding of the referred-to contents as well as the specification; their use of a betokening understanding of the attitude mode; their reliance on inter-level representational relations that are routes for preserving warrant; their immunity to brute error; their being warranted through understanding. (Burge 2013, p. 25).

This paper concludes that transparency models of self-knowledge do not have the resources to explain how we have *basic self-knowledge*, to employ Burge’s terminology. It thus raises some serious doubts as to whether transparency models can survive without being able to explain such a paradigmatic class of self-knowledge.

## Footnotes

1 I am following Burge’s notation according to which the underline indicates thoughts’ contents.

2 My characterization of the notion ‘privileged self-knowledge’ is deliberately general to be compatible with any model of self-knowledge that holds there is something epistemically special about self-knowledge. Although Burge himself does not use the term ‘privileged self-knowledge’, he understands that self-knowledge is special to the extent that it is direct, authoritative, and not merely empirical knowledge. Other models differ on the particular features of privileged self-knowledge. For example, an observational model holds that self-knowledge is privileged due to the privileged access to our minds via inner sense. In one version of the model, inner sense is a direct, complete and infallible mechanism of knowledge acquisition (Russell 1912). In another version, inner sense is rather strongly analogous to outer senses, and thus, it is not infallible. But it is still a privileged mechanism: other people do not have an ‘internal monitor or scanner’ of our minds (Armstrong 1968; Lycan 1996). In contrast, a constitutivist model rejects the idea that self-knowledge is achieved by a cognitive process that ‘discovers’ inner items. It actually denies that there are inner items to be discovered. Instead, constitutivism holds that we have privileged self-knowledge—i.e., authoritative self-knowledge—because we *constitute* our mental states when they are self-attributed (Wright 1989, 1998). Expressivism also denies the idea that self-knowledge is special because we have special access to inner mental items. However, in contrast to constitutivism, expressivism holds that the special aspect of self-knowledge relates to the fact that only the individual herself is in the position to express her mind, which confers her with first-person authority (Bar-On 2004; Finkelstein 2008). I explain transparency models in Sect. 3, in particular the versions advanced by Byrne—who understands privileged self-knowledge in terms of privileged and peculiar access—and Moran—who understands privileged self-knowledge in terms of deliberation. For a comprehensive guide to different models of self-knowledge see Gertler (2011a). For skeptical voices about privileged self-knowledge in general, and about substantial self-knowledge, see respectively Schwitzgebel (2012) and Cassam (2014).

3 Given the paper’s objective, I take for granted the idea that we have privileged self-knowledge of *cogito* judgments. In any case, to the best of my knowledge, there is no position which accepts that many of our mental self-ascriptions count as privileged self-knowledge but denies that *cogito* judgments are among such self-ascriptions.

4 Very roughly, his argument can be reformulated as follows. Knowledge of *cogito* judgments represents a paradigmatic case of privileged self-knowledge (i.e., basic self-knowledge). *Cogito*-judgments are self-verifying due to their self-reflexive form. Thus, the fact that the contents of one’s own thoughts are determined by factors external to the individual does not preclude the individual from having privileged self-knowledge of their *cogito*-judgments. In fact, being in a position to form such thoughts is evidence that the world has contributed to the formation of the person’s mind. Burge’s paper (1988) launched the so-called compatibilist debate between anti-individualism and self-knowledge.

5 See Ludlow and Martin (1998) for a good selection of early essays belonging to the compatibilist debate. A further question that goes beyond the scope of this paper is about the accuracy of Burge’s own characterization of *cogito *judgments. Since there is no established debate about this question, the paper relies on Burge’s account of this type of thought, which will be reconstructed throughout this section. Burge not only has coined the term, but he is also one of the few philosophers who dedicate a central place for *cogito* judgments within his general view of self-knowledge.

6 According to Burge, mental self-ascriptions that are not *cogito* judgments (for example, I believe that Paris is the capital of France) are by far the more common case. They also can amount to authoritative and privileged self-knowledge. However, non-*cogito* self-attributions are a different type of thought when compared to *cogito* judgments. Although instances of both can amount to privileged self-knowledge, *cogito* judgments seem to exhibit some other specific epistemic traits. For example, Burge holds, *cogito* judgments are self-verifying. I will return to this issue by the end of the current section.

7 Sawyer analyses the criticisms raised by Bernecker (1996), Gallois (1996) and Goldberg (2000) against Burge’s thesis that *cogito *judgments are self-verifying. Failure to distinguish between *cogito* and non-*cogito *judgments like (2) and (3) seems to be pervasive in such criticisms.

8 Note that simultaneity (i.e., thinking two thoughts at the same time) is not sufficient to think a thought through another.

9 The argument of this paper takes for granted the former thesis, but is neutral with respect to the latter.

10 According to Burge, *cogito* judgments have other epistemic characteristics such as being known in a non-inferential and non-empirical way.

11 See Sawyer (2002) for a defense of the thesis.

12 It could be argued that transparency models do not aim to give a unified explanation of privileged self-knowledge. Thus, apparently, such models would not be required to explain how *cogito* judgments count as cases of privileged self-knowledge. In fact, many of those who endorse the transparency model limit the application of that model to propositional attitudes—-and often, only to specific types of propositional attitudes. For example, Moran (2001, p. 10) holds that self-knowledge of sensations raises issues quite different from self-knowledge of other various kinds of attitudes. However, *cogito* judgments are relevantly similar to some types of propositional mental self-ascriptions such as of beliefs. Compare thoughts (2) and (3). Trait (iii) is the difference between them, but both can count as cases of privileged self-knowledge of propositional attitudes, respectively the one of judging and the one of believing. Even if a transparency model does not hold a unified explanation of self-knowledge, it is expected that the model would be able to handle both cases. The research question of the paper (i.e., whether transparency models can explain cases of *cogito* judgments) is in any case still relevant for investigating the explanatory limits of transparency models. See final section ‘Concluding Remarks’.

13 Throughout the paper, I will only use the term ‘transparency procedure’ to refer to this type of procedure, or process, of forming epistemically privileged mental self-ascriptions.

14
Cassam (2015) criticizes Byrne on a different basis. Like Byrne, Cassam holds that self-knowledge is acquired inferentially; however, Cassam holds that the transparency model is constructed upon the wrong inference.

15 In an earlier work, Byrne formulates the doxastic schema in terms of the following rule for self-ascribing beliefs: BEL: If *p*, believe that you believe that *p* (Byrne 2005, p. 95).

16 A similar point could be made concerning *reductio* arguments in which inference from a premise clearly does not entail belief in that premise. Only an inference from believed premises entails belief in the premise. Cassam (2015) raises some similar objections. In response, Byrne could insist that the doxastic schema takes place under special circumstances, and under such circumstances the inference is guaranteed to produce beliefs that are true. However, such circumstances are not specified in the schema. Setiya (2012) suggests that Byrne’s doxastic schema does not need to be understood in terms of an inference. Instead, it could be understood in terms of a different type of epistemic rule. Setiya claims that Byrne’s previous work (2005) supports that interpretation. However, Byrne himself talks in terms of inferential reasoning in his later work (2011a).

17 In arguing that *cogito* judgments do not involve any individual-level inference, Burge (2011, p. 184) does not deny that some ‘unconscious psychological causal processes’ connect second-order judgments about one’s mental occurrences and the first-order level mental occurrences.

18 Burge’s explanation is roughly the following: ‘Pure *cogito *thinkings are warranted through self-evidence. Individuals are warranted by understanding their thinkings. Such warrants yield immunity to *any *error. Warrant for impure *cogito *judgments lies in *de re *understanding. Understanding coordinates the lower level performative act with the higher-level *cogito *judgment. Impure *cogito *judgments are not immune to error.’ (Burge 2011, p. 210).

19 Another possible reply to the argument above would be to resist Burge’s suggestion that the very form of *cogito* judgments precludes that they are inferentially formed. I do not think this alternative is plausible, even if it is possible. However, my main argument developed in the next section does not depend on blocking this possibility.

20 As highlighted by O’Brien, although Moran provides some insightful ideas on self-knowledge, he does not seem to be explicit on the exact nature of his epistemic model. O’Brien (2003) suggests that the basis of Moran’s explanation is the ‘non-conceptual practical awareness manifest in the subject’s capacity to be led by reasons to judgment’ (O’Brien 2003, p. 381).

21 This understanding of the transparency procedure is based on Evans ’ original formulation (1982). However, some authors who support transparency models do not conceive beliefs as standing states. For example, Boyle (2009) conceives beliefs as mental ‘acts or activity’. I thank an anonymous reviewer for raising this point. Thus, the difficulty of explaining *cogito* judgments formulated in the above paragraph does not apply to such models. However, I raise further criticisms that do apply to such models. In the following paragraph, I formulate a criticism of the transparency procedure involving the self-ascription of occurrent thoughts. Arguably, this criticism could also apply to models that understand beliefs as occurrent thoughts. Additionally, my general criticism of transparency formulated towards the end of this section is independent of one’s conception of beliefs as standing states, occurrent thoughts, or enduring acts. See footnote 26 for more on Boyle’s interpretation of the transparency procedure.

22 See Byrne (2011b).

23 This is not to say that the application of the transparency procedure generates thoughts like the one that Paul has, in which he knows what he is thinking by reasoning about his appearance.

24
Byrne (2011b) formulates the alternative rule THINK (i.e., ‘If the inner voice speaks about *x*, believe that you are thinking about $$x')$$ in his attempt to explain privileged self-knowledge about what one is thinking. His main examples of THINK are, however, examples of passing thoughts, not of *cogito* judgments (i.e, ‘I am thinking’ is the conclusion of an inferential reasoning that takes a passing thought as evidence). Nevertheless, in any case, since THINK generates indirect, inferential knowledge, this version of the transparency procedure would also be subject to the kind of criticism advanced in Sect. 3.1.

25 Additionally, granted that the psychological form of *cogito* judgments makes them self-verifying, that same form also precludes them from being inferentially warranted.

26 This reading seems to be compatible with Boyle’s (2011) non-inferentialist interpretation of the transparency procedure which is based on the idea that ‘being in a given mental state M and believing oneself to be in M’ are not two distinct psychological conditions, but rather ‘two aspects of *one *cognitive state – the state, as we might put it, of knowingly believing $$p' $$(2011, p. 228). An alternative interpretation of Boyle’s position is that it actually offers skeptical doubts regarding *cogito* judgments as being a special class of thoughts.

27 Perhaps one would like to say that the transparency procedure, in its non-inferential reading, predicts that one self-ascribes a given mental state (and thereby has self-knowledge) *in* and *through* thinking about *p*. But to think something *in* a thought means that what is thought is part of the thought content. Thus, strictly speaking, a mental self-ascription cannot be a component of the first-order mental state that is ascribed. Self-ascribing the thought that *p* in thinking that *p* can only mean that the produced thought is about *p*. However, again, a *cogito* judgment is a judgment in which one thinks about *p* in and through thinking that one thinks about *p*.

28
Burge (2011) is one example of such a position.

29 There is little disagreement on the fact that they represent a small class of privileged self-knowledge, but being a small class does not imply that they are unimportant.
